# Oncology Clinicians’ Challenges to Providing Palliative Cancer Care—A Theoretical Domains Framework, Pan-Cancer System Survey

**DOI:** 10.3390/curroncol28020140

**Published:** 2021-04-09

**Authors:** Sharlette Dunn, Madelene A. Earp, Patricia Biondo, Winson Y. Cheung, Marc Kerba, Patricia A. Tang, Aynharan Sinnarajah, Sharon M. Watanabe, Jessica E. Simon

**Affiliations:** 1 Department of Community Health Sciences, Cumming School of Medicine, University of Calgary, Calgary, AB T2N 4Z6, Canada; sharlette.dunn1@ucalgary.ca (S.D.); ayn.sinnarajah@albertahealthservices.ca (A.S.); 2 Department of Oncology, Cumming School of Medicine, University of Calgary, Calgary, AB T2N 4N2, Canada; madalene.earp@ucalgary.ca (M.A.E.); pbiondo@ucalgary.ca (P.B.); winson.cheung@ucalgary.ca (W.Y.C.); Marc.Kerba@albertahealthservices.ca (M.K.); patricia.tang@albertahealthservices.ca (P.A.T.); 3 Department of Family Medicine, Cumming School of Medicine, University of Calgary, Calgary, AB T3M 1M4, Canada; 4 Department of Oncology, Faculty of Medicine and Dentistry, University of Alberta, Edmonton, AB T6G 1Z2, Canada; Sharon.Watanabe2@albertahealthservices.ca; 5 Department of Medicine, Cumming School of Medicine, University of Calgary, Calgary, AB T2N 2T9, Canada

**Keywords:** palliative care, oncology, theoretical domains framework

## Abstract

Despite the known benefits, healthcare systems struggle to provide early, integrated palliative care (PC) for advanced cancer patients. Understanding the barriers to providing PC from the perspective of oncology clinicians is an important first step in improving care. A 33-item online survey was emailed to all oncology clinicians working with all cancer types in Alberta, Canada, from November 2017 to January 2018. Questions were informed by Michie’s Theoretical Domains Framework and Behaviour Change Wheel (BCW) and queried (a) PC provision in oncology clinics, (b) specialist PC consultation referrals, and (c) working with PC consultants and home care. Respondents (*n* = 263) were nurses (41%), physicians (25%), and allied healthcare professionals (18%). Barriers most frequently identified were “clinicians’ limited time/competing priorities” (64%), “patients’ negative perceptions of PC” (63%), and clinicians’ capability to manage patients’ social issues (63%). These factors mapped to all three BCW domains: motivation, opportunity, and capability. In contrast, the least frequently identified barriers were clinician motivation and perceived PC benefits. Oncology clinicians’ perceptions of barriers to early PC were comparable across tumour types and specialties but varied by professional role. The main challenges to early integrated PC include all three BCW domains. Notably, motivation is not a barrier for oncology clinicians; however, opportunity and capability barriers were identified. Multifaceted interventions using these findings have been developed, such as tip sheets to enhance capability, reframing PC with patients, and earlier specialist PC nursing access, to enhance clinicians’ use of and patients’ benefits from an early PC approach.

## 1. Introduction

Patient symptom and quality of life outcomes are known to improve when palliative care (PC) is provided concurrently with cancer-modifying therapies [1,2]. Abrupt transitions from cancer-modifying treatments to palliative-focused care can cause unnecessary distress and suffering. For these reasons, clinical practice guidelines [3,4,5,6] increasingly recognize the integration of cancer care with PC as “best practice” for many patients with advanced cancer. Problematically, Canadian healthcare systems struggle to systematically provide early and integrated PC to meet the needs of the population [7,8]. For example, the provincial median for colorectal cancer patients to access PC was 53 days (IQR: 20–171 days) in 2014–2015 [9].

Providing early and integrated PC with cancer care is challenging for many reasons, including patients’ and families’ negative perceptions of PC [10], providers’ perceptions of PC [11,12], and the healthcare system’s siloed and fragmented organizational structure [13,14] The known factors impacting whether providers consider PC for patients include knowledge of available PC services, clarity and simplicity of referral processes, clinician time and competing priorities, communication issues, and role confusion within and between care teams [15,16].

As oncology clinicians regularly interact with advanced cancer patients, their influence on when and how PC is introduced is substantial. This study examined behavioural influences impacting when and how oncology clinicians refer advanced cancer patients to PC services. To accomplish this task, oncology clinicians across a provincial cancer care system were surveyed. Physicians, nurses, allied healthcare professionals (HCPs), and radiation therapists (RT) working with all cancer types were included, allowing robust assessments of their challenges, and response comparisons by professional role, cancer type, and cancer centre type.

## 2. Materials and Methods

### 2.1. Survey Instrument

A pilot study was conducted with gastrointestinal (GI) oncology clinicians [16]. The pilot study survey was built upon for this study through additional screening of the published academic literature for additional relevant concepts and questions [17,18]. Survey questions were mapped to Michie’s Behaviour Change Wheel (BCW) and Theoretical Domains Framework (TDF) [19,20,21]. These frameworks help identify sources of behaviour and provide starting points for devising behaviour change strategies.

The survey included 33 questions in five sections. Section 1 collected demographic information. Section 2, Section 3 and Section 4 queried challenges that oncology clinicians face: making referrals, working with PC teams, and addressing outpatients’ PC needs in the cancer clinic. Section 5 assessed the likelihood of recommending an early PC pathway, and if using early PC support would require substantial changes in practice.

Using a 7-point Likert scale, where 1 = “entirely disagree” and 7 = “entirely agree”, questions in Section 2, Section 3 and Section 4 were framed as follows: “for me, ‘X’ is a challenge” (i.e., 1 = “X” is not a challenge; 7 = ”X” is a challenge). Section 5 questions used the Likert scale but were not framed as “a challenge”. Lastly, 5 open-ended questions (1 per section) captured other challenges as well as ideas on better integrating PC into cancer care [22].

To address the likelihood of clinicians treating multiple cancer types, respondents were asked to choose one tumour type as their primary specialty when answering questions. The survey was pretested and refined using a “Think Aloud” strategy with 11 oncology clinicians [23]. Dillman’s guiding principles for internet surveys were also used to promote high response rates [24].

### 2.2. Survey Process

The survey was administered online using REDCap (v.7.2) from November 21, 2017, to January 31, 2018. All providers working in Cancer Care Alberta (CCA) cancer centres were targeted (*n* = 824). CCA, part of Alberta Health Services, oversees all cancer programs in the province. The survey link was emailed via provincial tumour group (i.e., groups of oncology clinicians with a specific tumour specialty) internal email distribution lists, and CCA’s comprehensive email distribution list of all oncology clinicians in Alberta, Canada. The survey link was also advertised in CCA’s internal online newsletter. Participation was voluntary, confidential, and anonymous. This study was approved by the Health Research Ethics Board of Alberta (HREBA.CC-17-0354).

### 2.3. Data Analysis

Survey questions using the Likert scale were analyzed by collapsing responses into three categories: 1–3 = low; 4 = neutral; 5–7 = high for ease of visualization and analysis. Responses of “don’t know” were included in data summaries but excluded from further analyses. Blank responses were excluded completely. Questions were ranked by the percentage of “high” responses, indicating agreement that the survey item was a challenge. To determine if respondent demographics were associated with survey responses, ordered logistic regression for each survey question was performed (using R version 3.5.1; package polr::MASS) [25]. Professional role, tumour specialty type, and cancer centre type were predictors in the model. The province’s tertiary centres were analyzed separately, because they have different palliative care access and norms of practice, while the smaller community centres were analyzed together. Interaction terms were not considered. One model was run for each survey question; statistical significance was set to *p* < 0.05 a priori. There was no adjustment for multiple *p*-value testing.

Open-ended questions were analyzed using conventional content analysis [26] by three researchers. Text fragments were inductively coded for primary themes by each researcher. Sections of text fragments were coded multiple times by each researcher to ensure inter-coder reliability. Final consensus on codes and the grouping of primary themes into overarching themes was achieved by all three coders.

## 3. Results

The overall response rate was 44% (366 respondents from an estimated 824 email recipients). Respondents were excluded from the final sample if they (1) reported never working with advanced cancer patients (*n* = 26), (2) only answered demographics questions (*n* = 43), or (3) reported a professional role that does not include usual outpatient care of cancer patients (*n* = 34: clerical *n* = 16; inpatient only *n* = 7; research only *n* = 6; clinical trials only *n* = 4; system analyst *n* = 1). The resulting sample size was 263.

### 3.1. Demographics

Forty-one percent of respondents were nurses, 25% were physicians, and 18% were allied HCPs (Table 1).

Medical oncology was the primary oncological discipline practiced followed by radiation oncology. Two thirds of respondents work in one of Alberta’s tertiary cancer centres in Calgary or Edmonton. Perspectives on many different tumour specialties were received, including breast (19%), palliative care (16%), gastrointestinal (14%), lung (14%), hematological (11%), and head and neck (8%) tumour specialties. In Alberta, the palliative care tumour specialty focuses on providing specialist PC and/or oncology care to PC patients (Table 1). Most respondents had ≥10 years of experience in their professional role (59%), and 55% reported caring for patients with advanced cancer “most of the time”.

### 3.2. Challenges in Providing PC

**Figure 1:**
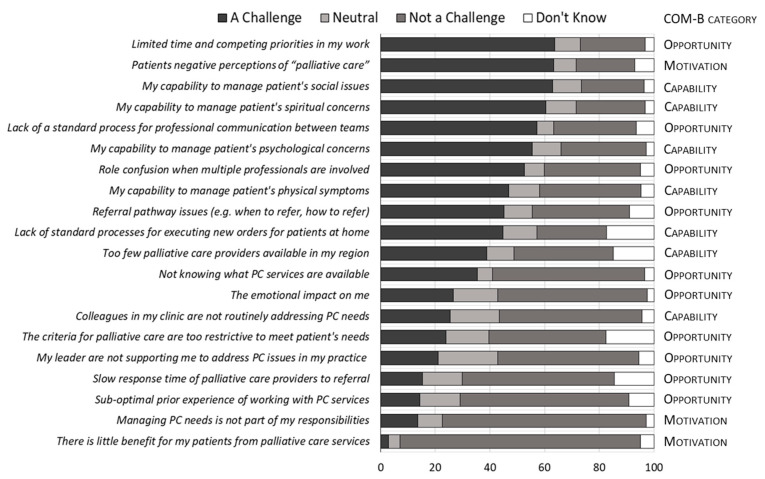
Oncology clinicians most frequently identified challenges to early, systematic, oncology-integrated palliative care for advanced cancer patients. Survey questions were posed using an ordinal scale (1–7) and framed as follows: “a challenge I face is:”. All agree responses (entirely = 7; mostly = 6; somewhat = 5) were collapsed as “challenge”. All disagree responses (entirely = 1, mostly = 2; somewhat = 3) were collapsed as “not a challenge”. Neither agree nor disagree responses were labelled “neutral” = 4. Survey questions are ranked by the percentage of observed “challenge” responses (largest to smallest). Questions are mapped to Michie’s Capability, Opportunity, and Motivation (COM) Behaviour (COM-B) Change Wheel.

Figure 1 illustrates the challenges clinicians face in providing earlier, more integrated PC, ranked from those most frequently cited as a challenge to those least frequently cited. The three most frequently identified challenges were as follows: (1) limited time and competing priorities (64%), (2) patients’ negative perceptions of PC (63%), and (3) clinicians’ own capability to manage patients’ social issues, such as living alone (63%).

Figure 1 also maps each challenge to the three components of the BCW: opportunity, motivation, and capability. Conversely, factors rarely identified as challenging included beliefs in the benefit of PC to patients, and in providing PC as part of oncology clinicians’ roles, each mapping to the motivation domain of BCW [21].

Lastly, 81% of clinicians are likely to recommend early PC to their patients. When asked if substantial changes to their practice would be required to use earlier PC, 53% of clinicians “agreed” they would, 31% “disagreed” they would, and the remainder responded, “don’t know”.

### 3.3. Clinician Characteristics and Challenge Perceptions

Factors (professional role, tumour lens, and work location) associated with survey respondents identifying aspects of earlier use of palliative care as being “challenging” to them, for the five most frequently identified challenges, are reported in Table 2.

#### 3.3.1. Professional Role

Professional role was often a significant predictor in the perception of something being “a challenge”. However, the survey question regarding “limited time and competing priorities” (the top-ranked challenge) did not elicit significantly different responses by professional role (Table 2). For the second ranked challenge, “patients’ negative perceptions of PC”, relative to physicians (of whom ~50% agree), nurses (75% agree), allied HCPs (73% agree), and RTs (83% agree), were all significantly more likely to agree that this is a challenge (odds ratios (ORs): 3.4–6.9). With regard to managing patients’ social issues (third rank), nurses and allied HCPs (50–65% agree) were significantly less likely than physicians (77% agree) to agree that this was a challenge (OR_Nurses_ = 0.18, 95%CI = 0.18 to 0.82; OR_Allied HCP_ = 0.18, 95%CI = 0.07 to 0.46). For patients’ spiritual issues (fourth rank), only allied HCPs differed significantly in their response from physicians. Only 49% of allied HCPs agreed that this is a challenge, versus 64% of physicians. Allied HCPs include those practicing social work, psychology, and spiritual care, which could explain this difference. Finally, compared to physicians (42% agree), all other providers groups were more likely to agree (67–70% agree) that a “lack of standard process for professional communication between teams” (fifth ranked) was a challenge (OR_Nurses_ = 3.6, 95%CI = 1.82 to 7.29; OR_Allied HCP_ = 4.53, 95%CI = 1.86 to 11.6).

#### 3.3.2. Practice Location

Practice location was associated with survey response for three of the five top-ranked challenges. Respondents located at the tertiary cancer centre in Edmonton were less likely to agree that patients’ negative perception of PC is a challenge (64% agree), as compared to respondents at Calgary’s tertiary centre (71% agree). Respondents located at community cancer centres or other non-tertiary sites were more likely to agree (51% vs. 63%) that lack of standard process for communication between teams was a challenge (compared to respondents located at Calgary’s tertiary cancer centre) (Table 2).

#### 3.3.3. Tumour Specialty

Tumour specialty was not associated with survey response for any of the five top-ranked challenges (Table 2). Note that GI is the “reference” group to allow comparison to a previously described tumour specialty [16], and survey results for hematology are published elsewhere [27].

### 3.4. Content Analysis

**Figure 2:**
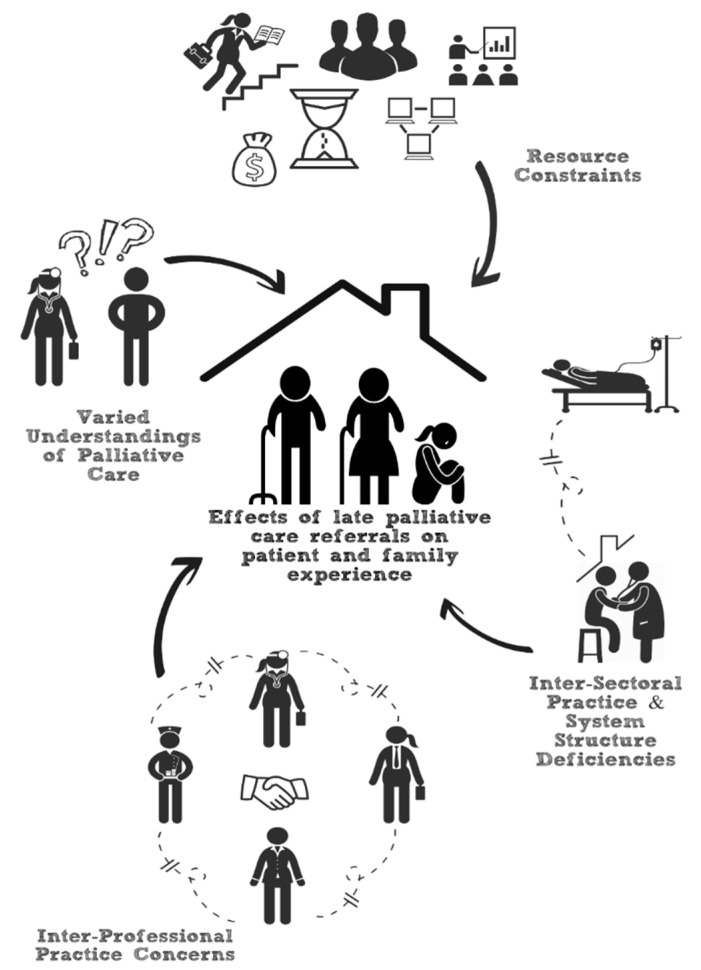
A visual framework depicting the relationships between the overarching themes (barriers to PC) identified in the content analysis of open-ended survey questions.

Results from the content analysis provide further context and allow a deeper understanding of the challenges faced by oncology clinicians in integrating PC into their practice. Responses to at least one open-ended question were provided by 112 participants. Four overarching themes emerged: (1) patients’ varied perceptions of PC, (2) inter-professional practice challenges, (3) inter-sectoral practice challenges, and (4) resource constraints (Figure 2).

#### 3.4.1. Patients’ Varied Perceptions of PC

A hematology nurse observed the following: “[The] main challenge is not referring soon enough; generally, because of patients’ negative perceptions (e.g., palliative care means I’m dying or we’re giving up)”. “Normalizing PC to remove the stigma associated with it” and providing “more education for patients and staff” would help better integrate PC and cancer care. A similar sentiment was expressed by a social worker working with CNS-related cancers: “patients fear that referral to PC means the system and care team are giving up; [it’s] almost seen as a hit to their hope”.

#### 3.4.2. Inter-Professional Practice Challenges

The logistical challenges of inter-professional practice were noted by several oncology clinicians, particularly around the scope of practice, communication, and role confusion. As one palliative-focused nurse summarized, when “there are multiple professionals involved, [it] can be a bit overwhelming trying to figure out who to contact to provide what”. Echoing this, a GI physician commented that it can be difficult to know “who is the primary physician in charge of dealing with ordering tests, following up on tests and/or admitting patients”.

#### 3.4.3. Inter-Sectoral Practice Challenges

Challenges in inter-sectoral practice, often arising due to system structure, were described. Sources of friction included the following: referral processes and criteria, continuity of care, and how patients navigate the system. Interactions between cancer centres and urban-based palliative home care were often cited as challenging. In a statement echoed across sites and tumour specialties, a tertiary cancer centre nurse commented, “Home care is refusing to send on referrals that are for PC unless a patient is symptomatic. We are getting push back trying to get end of life care if the patient does not have an obvious symptom besides advanced life ending cancer”. A hematology nurse in a tertiary cancer centre noted the following:

*“Our patients are reliant on transfusion support until the very last days of their life [.] PC practitioners will often refuse to see patients until these transfusions are discontinued, making the time for support incredibly short... there is a lack of understanding from PC practitioners that although these patients might have failed treatment, they may still have good quality of life for a period of time with supportive care.”*

This statement is echoed by clinicians specializing in head and neck tumours who experience similar difficulties in that “some patients do not fit into the specific need parameters that PC has”. Physicians treating patients with these cancers noted challenges with “transfer of care versus shared care”. Relevant to continuity of care, another recurring theme involved problems arising from the “doctor of the day” situation, as noted by a physician in a cancer centre.

*“[A] large component of the benefit from PC Services… is the relationship that develops between the care provider and patient. Under the current system, patients and families can meet different PC providers in hospital, in clinic (and this may vary week to week in clinic), and in the community. I feel the lack of continuity is a huge drawback of the current system.”*

#### 3.4.4. Resource Constraints

Resource constraints, including time, space, staff, education, training, and accessibility, were raised frequently. According to a clinical associate in a tertiary cancer centre, “lack of time in very busy clinics is by far the biggest barrier”. A physician in a tertiary cancer centre explains, “I almost always reach my maximum capacity for follow up clinics many weeks ahead and cannot usually book follow up appointments to specifically address most of these issues”.

## 4. Discussion

The most frequently perceived challenges in providing PC concurrently with cancer-modifying therapies are limited time and competing priorities, concerns about patients’ negative perceptions of PC, and clinicians’ own capability to manage patients’ social issues. These mapped to all three domains of the Capability, Opportunity, and Motivation (COM-B) model. This coverage suggests a multifaceted and potentially complex approach is required to address identified barriers, using multiple intervention functions from the BCW.

Oncology clinicians, across profession, tumour specialty group, and location, agreed these are key challenges. The results are also largely consistent with those reported in earlier studies, including the pilot study [16]. Time and competing priorities were the top-ranked barriers in the pilot [16], which is confirmed by the current study and others [28,29,30]. In prior reports, negative perceptions of PC were also often a cited challenge [10,29]. Renaming or reframing the meaning have been suggested as potential strategies to address negative perceptions of PC. [31,32,33]. Interestingly, adding to these known challenges, we found that professional role is a factor, with concerns about patients’ negative perceptions of PC perceived as an even greater barrier by nurses, allied healthcare professionals, and radiation therapists than physicians. This result may reflect differences in professional roles and responsibilities, levels of training and education around PC conversations, or prevailing attitudes and perceptions by profession. Planned interventions may need to take these variations into consideration.

This study queried clinicians’ perceived capability to manage patients’ social, spiritual, psychological, and physical needs, and these factors were identified as barriers by 65%, 63%, 57%, and 49% of clinicians, respectively. These results are consistent with a recent study of 649 radiation oncologists, 66%, 51%, and 48% of whom were not confident in their ability to manage patients’ depression, anxiety, or assess psychosocial issues, respectively [30]. Relative to physicians, nurses and HCPs were more likely to perceive themselves as capable of managing patients’ psychosocial issues, particularly social issues, further highlighting differences in attitudes and capacity by professional role. These differences should also be considered when planning interventions. The predominant pattern for professional role was for non-physician professional roles to identify challenges more often than physicians. It is not clear if this reflects differences in attitudes and perceptions, or the realities of different professional roles.

Considering tertiary and community cancer centre differences, the lack of PC services available in community cancer centres may paradoxically have a positive impact on perceived role clarity and communication between teams, as there are fewer providers involved in a patient’s care [32,34].

### 4.1. Next Steps

The findings of this study have informed the development of a guideline and pathway to better integrate palliative and cancer care [35]. In response to the opportunity gap of lack of time and competing priorities (challenge #1), environmental restructuring and enablers, such as a dashboard and cueing processes, was created to assist oncologists in rapidly screening upcoming clinic patients for advanced cancer and unmet symptom and PC referral needs.

To create a shared understanding and a positive clinician perception of how patients perceive PC (challenge #2), education, persuasion, and modeling were used, including the following: language and phrasing tips for clinicians to help them introduce palliative care as an added layer of support; “Palliative care myth busting slides” on the waiting room TVs and a palliative care nurse specialist repeatedly visiting clinics and modeling a palliative care approach. Enhancing clinician capability to manage patients’ social issues (challenge #3) can include education, but we also created an integrated EMR “shared care letter” to improve care coordination with family physicians. This helps teams optimally use cancer centre and community resources to address these issues, and includes guidance on referrals to psychosocial and/or PC providers for more complex needs.

### 4.2. Strength and Limitations

A strength of this study is that all clinicians working in a large, population-based cancer system were surveyed compared to prior studies that have only considered physician perspectives, or a single centre. Further, this survey was informed by evidence-based theoretical models to comprehensively assess behavioural factors that other surveys have not explored. Exploring the potential differences amongst professions, tumour types, and centres helped indicate which intervention functions might need to be customized for specific clinician groupings.

There are several limitations to the current study. First, the sample response rate is an estimate. As the total number of oncology clinicians working in the province with patients with advanced cancer is unknown, the response rate may be an underestimation. Second, although the distribution of oncology clinicians’ responses by professional role, tumour specialty groups, and location was largely representative of those employed by the cancer system, it was unbalanced. For example, physicians from the Calgary tertiary centre and nurses with a GI tumour specialty were represented more than would be expected by chance. This distribution suggests sampling bias; thus, response averages should be interpreted with caution. Third, as no adjustment was made for multiple *p*-value testing, some of the findings need to be explored further and replicated in other settings.

## 5. Conclusions

This provincial survey of oncology clinicians indicates the main barriers to providing PC concurrently with disease-directed therapies included concerns about patients’ negative perceptions of PC, as well as opportunity and capability concerns. The knowledge of these complex barriers is being used to design multifaceted interventions, including normalizing descriptions of PC in patient-directed materials, clarification of referral criteria to PC services, communication templates between PC and oncology providers, and semi-automated screening of patients to identify those who may benefit from referral [22].

## Figures and Tables

**Table 1:** Demographics of survey respondents.

Variable		*n* (%)
**All Respondents**		263 (100%)
Primary role	Nurse	109 (41)
	Physician	65 (25)
	Allied healthcare professional	48 (18)
	Radiation Therapist	28 (11)
	Administration ^1^	8 (3)
	Educator/Facilitator	5 (2)
Primary location	Tertiary centre—Edmonton	78 (30)
	Tertiary centre—Calgary	99 (38)
	Community centre/Other ^1^	86 (33)
Primary Oncological Discipline	Medical Oncology	128 (49)
	Radiation Oncology	55 (21)
	Surgical Oncology	7 (3)
	Other Oncology Disciplines ^2^	10 (4)
	Not applicable ^3^	63 (24)
Tumour lens	Breast	51 (19)
	Palliative care	42 (16)
	Gastrointestinal	37 (14)
	Lung	36 (14)
	Hematological	30 (11)
	Head and Neck	20 (8)
	Gynecological	16 (6)
	Genito-urinary	14 (5)
	Neurological	7 (3)
	All cancers	6 (2)
	Other cancers ^4^	4 (2)
Work with advanced cancer patients	Most of the time	145 (55)
	Sometimes	108 (41)
	Rarely	10 (4)
Gender	Female	210 (80)
	Male	52 (20)
	Not Reported	1 (0)
Years in role	≥10 years	155 (59)
	<10 year	108 (41)

Administration includes managers and leaders who are included as they set policies on palliative care (PC) access for their centres. Their experiences and beliefs are important to assess when changing practice. ^1^ This category includes Jack Ady CC (*n* = 20), Grande Prairie Community Centre (*n* = 13), Central Alberta Community Centre (*n* = 13), Margery E. Yuill Community Centre (*n* = 11), other community centres (*n* = 18), and other non-community centre locations (*n* = 11). ^2^ This category includes gynecological oncology (*n* = 6) and psychosocial oncology (*n* = 4). ^3^ Primary oncological discipline was “not applicable” for respondents who work with patients treated by any or all oncological disciplines. ^4^ Cutaneous (*n* = 1), endocrine (*n* = 1), pediatric (*n* = 1), sarcoma (*n* = 1).

**Table 2:** Factors (professional role, tumour lens, and work location) associated with survey respondents identifying aspects of earlier use of palliative care as being “challenging” to them, for the five most frequently identified challenges.

		Count (%) Who “Agree” Is a Challenge; OR (95% CI)														
		Professional Role					Tumour lens							Location		
	Question	Physician	Nurse	Allied HCP ^1^	RT	Other ^2^	GI	Lung	Breast	Blood	H&N	Palliative	Other ^3^	Tertiary—Calgary	Comm./ Other	Tertiary—Edmonton
1	Limited time and competing priorities in my work.	*41/64 = 64%; ref.*	73/104 = 70%; 1.14 (0.56–2.3)	27/43 = 63%; 0.79 (0.33–1.93)	14/21 = 67%; 1.34 (0.46–4.11)	5/10 = 50%; 0.91 (0.26–3.44)	*26/37 = 70%; ref.*	28/34 = 82%; 2.07 (0.67–6.97)	28/47 = 60%; 0.75 (0.29–1.93)	18/28 = 64%; 0.85 (0.28–2.57)	12/18 = 67%; 0.89 (0.24–3.5)	24/36 = 67%; 0.87 (0.29–2.57)	24/42 = 57%; 0.62 (0.22–1.71)	*54/87 = 62%; ref.*	55/84 = 65%; 1.19 (0.59–2.4)	51/71 = 72%; 1.55 (0.75–3.23)
2	Patients have negative perceptions of “palliative care”.	*32/64 = 50%; ref.*	**79/105 = 75%; 3.4 (1.68–7.03)**	**33/45 = 73%; 3.46 (1.44–8.7)**	**15/18 = 83%; 6.93 (1.82–34.97)**	8/12 = 67%; 2.75 (0.76–11.7)	*26/36 = 72%; ref.*	23/33 = 70%; 0.96 (0.32–2.9)	34/45 = 76%; 1.35 (0.46–3.95)	20/26 = 77%; 1.38 (0.4–5.06)	15/20 = 75%; 1.64 (0.41–7.18)	28/41 = 68%; 0.81 (0.27–2.43)	21/43 = 49%; 0.47 (0.16–1.32)	*64/90 = 71%; ref.*	57/81 = 70%; 0.63 (0.29–1.35)	**47/73 = 64%; 0.43 (0.2–0.91)**
3	My capability to manage patients’ social issues (e.g., lives alone).	*49/64 = 77%; ref.*	**68/105 = 65%; 0.39 (0.18–0.82)**	**21/42 = 50%; 0.18 (0.07–0.46)**	16/21 = 76%; 0.82 (0.24–3.03)	4/9 = 44%; 0.28 (0.07–1.12)	*27/37 = 73%; ref.*	24/34 = 71%; 1.11 (0.37–3.38)	29/45 = 64%; 0.89 (0.32–2.42)	17/28 = 61%; 0.8 (0.26–2.48)	12/18 = 67%; 1.1 (0.28–4.46)	24/36 = 67%; 0.76 (0.24–2.38)	25/43 = 58%; 0.61 (0.21–1.74)	*53/87 = 61%; ref.*	57/82 = 70%; 2.09 (1–4.43)	48/72 = 67%; 2.05 (1.01–4.25)
4	My capability to manage patients’ spiritual concerns (e.g., meaning of life).	*41/64 = 64%; ref.*	68/105 = 65%; 0.79 (0.39–1.55)	**21/43 = 49%; 0.31 (0.13–0.73)**	18/21 = 86%; 3.16 (0.87–15.26)	3/9 = 33%; 0.51 (0.14–1.86)	*23/37 = 62%; ref.*	25/34 = 74%; 2.49 (0.87–7.49)	27/46 = 59%; 1.21 (0.48–3.07)	17/28 = 61%; 1.51 (0.52–4.44)	13/18 = 72%; 3.49 (0.94–14.5)	22/36 = 61%; 1.03 (0.36–2.97)	24/43 = 56%; 1.06 (0.4–2.81)	*50/87 = 57%; ref.*	**55/83 = 66%; 2.17 (1.07–4.47)**	46/72 = 64%; 1.53 (0.76–3.13)
5	Lack of standard processes for professional communication between teams.	*27/64 = 42%; ref.*	**70/104 = 67%; 3.59 (1.82–7.29)**	**30/43 = 70%; 4.53 (1.86–11.58)**	13/19 = 68%; 3.26 (1.02–11.35)	8/12 = 67%; 3.5 (0.93–15.32)	*23/37 = 62%; ref.*	19/33 = 58%; 0.7 (0.25–1.96)	25/42 = 60%; 0.96 (0.36–2.55)	17/28 = 61%; 0.68 (0.22–2.09)	14/19 = 74%; 1.41 (0.36–5.91)	28/39 = 72%; 1.22 (0.41–3.68)	22/44 = 50%; 0.47 (0.17–1.3)	*55/87 = 63%; ref.*	**41/80 = 51%; 0.36 (0.17–0.76)**	52/75 = 69%; 0.73 (0.34–1.52)

Count (%) denominator and regression models do not include respondents who responded, “Don’t Know” or “N/A” to a survey question. Ordered logistic regression for each survey question was performed (response modelled as “low” Likert scale scores 1–3 [reference], “neutral” Likert Scale score 4, “high” Likert scale score 5–7, for identify something as “a challenge”) with professional role, tumour speciality type (lens), and location included as predictors in the model. One model was run for each survey question. HCP=Health Care Providers; RT=Radiation Therapists; GI=Gastrointestinal; H&N=Head and Neck. ^1^ Allied HCP roles: pharmacy, social work, psychology, physiotherapy, occupational therapy, and spiritual care. ^2^ Other professional roles: administrators, educators, and facilitators. ^3^ The “Other” category includes tumour lens, gynecological, genitourinary, neurological, all cancer, cutaneous, endocrine, pediatric, sarcoma.

## Data Availability

The datasets generated for this study are available from the corresponding author on reasonable request.
